# Impact of flow and temperature on patient comfort during respiratory support by high-flow nasal cannula

**DOI:** 10.1186/s13054-018-2039-4

**Published:** 2018-05-09

**Authors:** Tommaso Mauri, Alessandro Galazzi, Filippo Binda, Laura Masciopinto, Nadia Corcione, Eleonora Carlesso, Marta Lazzeri, Elena Spinelli, Daniela Tubiolo, Carlo Alberto Volta, Ileana Adamini, Antonio Pesenti, Giacomo Grasselli

**Affiliations:** 1 Anesthesia and Critical Care, Department of Pathophysiology and Transplantation, University of Milan, Via F. Sforza 35, 20122 Milan, Italy; 2 Department of Anesthesia, Critical Care and Emergency, Fondazione IRCCS Ca’ Granda Ospedale Maggiore Policlinico, Via F. Sforza 35, 20122 Milan, Italy; 3 Department of Morphology, Surgery and Experimental Medicine, Section of Anesthesia and Intensive Care, University of Ferrara, Ferrara, Italy

**Keywords:** High-flow nasal oxygen, Spontaneous breathing, Acute hypoxemic respiratory failure, Patient comfort, Nursing

## Abstract

**Background:**

The high-flow nasal cannula (HFNC) delivers up to 60 l/min of humidified air/oxygen blend at a temperature close to that of the human body. In this study, we tested whether higher temperature and flow decrease patient comfort. In more severe patients, instead, we hypothesized that higher flow might be associated with improved comfort.

**Methods:**

A prospective, randomized, cross-over study was performed on 40 acute hypoxemic respiratory failure (AHRF) patients (PaO_2_/FiO_2_ ≤ 300 + pulmonary infiltrates + exclusion of cardiogenic edema) supported by HFNC. The primary outcome was the assessment of patient comfort during HFNC delivery at increasing flow and temperature. Two flows (30 and 60 l/min), each combined with two temperatures (31 and 37 °C), were randomly applied for 20 min (four steps per patient), leaving clinical FiO_2_ unchanged. Toward the end of each step, the following were recorded: comfort by Visual Numerical Scale ranging between 1 (extreme discomfort) and 5 (very comfortable), together with respiratory parameters. A subgroup of more severe patients was defined by clinical FiO_2_ ≥ 45%.

**Results:**

Patient comfort was reported as significantly higher during steps at the lower temperature (31 °C) in comparison to 37 °C, with the HFNC set at both 30 and 60 l/min (*p* < 0.0001). Higher flow, however, was not associated with poorer comfort.

In the subgroup of patients with clinical FiO_2_ ≥ 45%, both lower temperature (31 °C) and higher HFNC flow (60 l/min) led to higher comfort (*p* < 0.01).

**Conclusions:**

HFNC temperature seems to significantly impact the comfort of AHRF patients: for equal flow, lower temperature could be more comfortable. Higher flow does not decrease patient comfort; at variance, it improves comfort in the more severely hypoxemic patient.

## Background

The high-flow nasal cannula (HFNC) is a new non-invasive, easy-to-use respiratory support for adult patients with acute hypoxemic respiratory failure (AHRF). The HFNC delivers 30–60 l/min of humidified air and oxygen blend at the desired FiO_2_ and temperature [1].

Several recent randomized clinical trials in patients with or at risk for AHRF described decreased need for invasive mechanical ventilation and improved survival by early application of HFNC compared to standard oxygen or non-invasive positive pressure ventilation (NIPPV) [2, 3]. The HFNC is associated with several beneficial physiologic effects, potentially promoting spontaneous breathing, avoiding patient exhaustion, and decreasing the risk of patient self-inflicted lung injury (P-SILI) [4–8].

Although most studies reported that application of the HFNC is associated with higher patient comfort in comparison to NIPPV [2, 3], none investigated the degree of comfort at different HFNC settings. Improved comfort might be a strong clinical endpoint per se [9, 10] and useful to guide optimal HFNC settings, which are still debated. Previous clinical studies used highly heterogeneous criteria to set flow and temperature [11–15], and a study from our group described that physiologic effects of HFNC in AHRF patients might be maximized by different personalized flow rates rather than simply by the highest value [8], but no study investigated the effects of different temperature settings (with highest value commonly considered as optimal).

In this collaborative project, nurses assessed comfort during HFNC at increasing flow and temperature in AHRF patients. Our hypothesis was that higher flow and temperature might reduce comfort. We also planned a subanalysis to describe whether, in the subgroup of more severe patients clinically requiring higher inspiratory oxygen fraction (FiO_2_), higher flow could result in improved comfort. The rationale was that higher HFNC flow more effectively corrects hypoxia and copes with elevated inspiratory effort [7, 8], potentially tampering the neural drive and improving comfort.

## Methods

### Study population

Non-intubated AHRF patients admitted to the intensive care unit (ICU) of the Fondazione IRCCS Ca′ Granda Ospedale Maggiore Policlinico, Milan, Italy were included. Inclusion criteria were: new/worsening respiratory symptoms (e.g., dyspnea) following a known clinical insult (e.g., pneumonia) within 1 week; PaO_2_/FiO_2_ ≤ 300 despite additional oxygen as per clinical decision; and evidence of pulmonary infiltrates on chest X-ray or CT scan.

Exclusion criteria were: age < 18 years; tracheostomy; hemodynamic instability (hypotension with mean arterial pressure < 60 mmHg despite adequate volume resuscitation and vasoactive drugs); AHRF only due to cardiac failure; severe chronic obstructive pulmonary disease (i.e., documented stage IV or patients prescribed with home oxygen); and altered mental status.

The Fondazione IRCCS Ca′ Granda Ethical Committee approved the study (reference 193_2017), and informed consent was obtained from each patient according to local regulations. Four ICU nurses (AG, FB, LM, and IA) independently performed the enrolment, study protocol, and data collection. Part of the data reported here has been presented in the form of an abstract awarded with the Nurses and Allied Healthcare Professionals Award of the European Society of Intensive Care Medicine (ESICM Congress, Wien, Austria, October 2017).

### Demographics and clinical severity

At enrolment, the participants’ age, sex, presence of bilateral infiltrates, Sepsis-related Organ Failure Assessment (SOFA) score, AHRF etiology, PaO_2_/FiO_2_ ratio, Simplified Acute Physiology Score (SAPS) II at ICU admission, and body mass index (BMI) were collected.

### Continuous monitoring

Three-lead electrocardiogram, peripheral arterial oxygen saturation, and invasive arterial pressure were monitored during the whole study protocol.

### Study protocol

Patients were nonsedated and kept in a semirecumbent position in a calm environment. Each patient underwent, in random order, four 20-min steps:

1. Gas flow 30 l/min and temperature 31 °C (HF30-T31).
2. Gas flow 60 l/min and temperature 31 °C (HF60-T31).
3. Gas flow 30 l/min and temperature 37 °C (HF30-T37).
4. Gas flow 60 l/min and temperature 37 °C (HF60-T37).

The HFNC was inserted through specific medium/large nasal prongs (Fisher & Paykel Healthcare, Auckland, New Zealand) to fit the nares size. The attending physician clinically chose the FiO_2_ before enrolment to target peripheral saturation of 92–98% on pulse oximetry. FiO_2_ was continuously measured by a dedicated system (AIRVO™ 2; Fisher & Paykel Healthcare, Auckland, New Zealand) connected to the HFNC and kept constant during all phases by adjusting the additional oxygen wall supply. The system can deliver airflows between 30 and 60 l/min with set FiO_2_ between 0.21 and 1.0. To identify the subgroup of more severe patients, a threshold for clinically selected FiO_2_ ≥ 45% was chosen, as this represented a viable compromise between the need for excluding less severe patients (i.e., those left at FiO_2_ ≤ 40% by the attending physician) [16] and assuring adequate subgroup numerosity.

### Measures

Toward the end of each study phase, we collected data for oxygen saturation, FiO_2_, systolic and diastolic arterial pressure, heart rate, Borg dyspnea score, respiratory rate, and comfort score by Visual Numerical Scale (VNS) ranging between 1 (extreme discomfort) and 5 (very comfortable). In fact, the VNS is commonly used to assess comfort in the clinical environment [17], and has already been used in larger clinical studies on HFNC (e.g., FLORALI study [2]).

### Statistical analysis

The enrolment of 40 patients was planned (study power = 0.8, α = 0.05) based on a clinically meaningful difference of 2.0 ± 1.5 points in patient comfort between HF30-T31 vs HF60-T37 [1]. Numerosity seemed reasonable also to obtain sufficiently large subgroups.

Data are expressed as mean ± standard deviation or median (interquartile range (IQR)).

Differences between variables measured during the four study phases (HF30-T31 vs HF60-T31 vs HF30-T37 vs HF60-T37) in the whole population and in the two FiO_2_ range subgroups (fixed effects) were tested by a generalized linear mixed model (GLIMMIX) with a carryover effect. Post hoc Bonferroni or Tukey multiple comparison tests were performed. The carryover variable was the combination of flow and temperature applied in the preceding phase to exclude influence from the random sequence.

For comparisons of baseline variables between groups defined by a FiO_2_ threshold of 45%, a paired *t* test or Wilcoxon’s signed rank test was used as appropriate. Baseline categorical variables between the two subgroups were compared by chi-square test.

The study phase associated with the highest VNS value for comfort was indicated as “best comfort settings” for that individual patient, while the one with the lowest value was indicated as “worst comfort settings”.

*p* < 0.05 (two-tailed) was considered statistically significant. The statistical software used was SigmaPlot 12.0 (Systat Software Inc., San Jose, CA USA) and SAS 9.2 (SAS Institute Inc., Cary, NC, USA).

## Results

### Study population

Forty patients were enrolled: mean age was 57 ± 15 years and 16 were female (40%). The clinical condition was quite severe, with SAPS II of 36 ± 9 and median SOFA score of 4 (IQR 4–6). Pulmonary infiltrates were bilateral in 28 cases (70%). AHRF was of pulmonary origin in 48% and of infectious etiology in 56% of the patients. The main characteristics of the study population are presented in Table 1.

**Table 1:** Baseline characteristics of the study population

Characteristic	Overall population (*N* = 40)	FiO_2_ < 45% (*N* = 24)	FiO_2_ ≥ 45% (*N* = 16)	*p* value, FiO_2_ < 45% vs FiO_2_ ≥ 45%
Age (years)	57 ± 15	55 ± 14	62 ± 16	0.17
Female sex, *n* (%)	16 (40%)	8 (33.3%)	8 (50%)	0.29
Monolateral/bilateral infiltrates, *n* (%)	12 (30%)/28 (70%)	8 (33%)/16 (67%)	4 (25%)/12 (75%)	0.57
SOFA score	4 (3–6)	3.5 (3–6)	4 (3–6)	0.89
Pulmonary/extrapulmonary cause of ARDS, *n* (%)	19 (48%)/21 (53%)	11 (46%)/13(54%)	8 (50%)/8 (50%)	0.80
Infectious/non-infectious cause of ARDS, *n* (%)	22 (56%)/18 (45%)	14 (58%)/10 (42%)	8 (50%)/8 (50%)	0.60
FiO_2_	40 (40–50)	40 (40–40)	50 (50–60)	< 0.0001*
Respiratory rate (bpm)	22 (18–24)	20 (18–24)	24 (20–30)	0.05*
Borg scale	2 (1–3)	1 (0–3)	3 (2–4)	0.02*
Comfort	4 (3–5)	4 (3–5)	4 (3–5)	0.81
SpO_2_/FiO_2_	241 (196–249)	248 (243–250)	192 (161–199)	< 0.0001*
SAPS II	36 ± 9	38 ± 8	35 ± 11	0.35

Normally distributed variables reported as mean ± standard deviation, non-normal variables reported as median (interquartile range). *p* values obtained by *t* test, Wilcoxon rank-sum test, or chi-square test as appropriate

*ARDS* acute respiratory distress syndrome, *FiO*_*2*_ inspired oxygen fraction, *SAPS* Simplified Acute Physiology Score, *SOFA* Sepsis-related Organ Failure Assessment, *SpO*_*2*_ peripheral oxygen saturation

*Data significant at *p* < 0.05

### Effects of flow and temperature on comfort in the study population

**Fig. 1:**
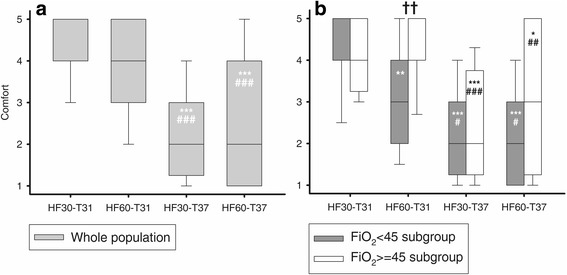
Impact of temperature and flow on patient comfort. Values reported as median with 10°, 25°, 75°, and 90° percentiles as vertical boxes with error bars. **a** Whole population, overall *p* < 0.0001 (GLIMMIX). Post hoc analysis: ***p < 0.001 vs HF30-T31, ###*p* < 0.001 vs HF60-T31. **b** Subgroup analyses: grey boxes represent FiO_2_ < 45% subgroup, while white boxes represent FiO_2_ ≥ 45% subgroup. *p* values from GLIMMIX analysis: treatment effect *p* < 0.001; FiO_2_ effect = 0.055; interaction = 0.035. Post hoc analysis: *p < 0.05 vs HF30-T31; **p < 0.01 vs HF30-T31; ***p < 0.001 vs HF30-T31; #*p* < 0.05 vs HF60-T31; ##*p* < 0.01 vs HF60-T31; ###*p* < 0.001 vs HF60-T31; ††*p* < 0.01 vs FiO_2_ < 45% subgroup (same step). *FiO*_*2*_ inspired oxygen fraction, *HF30-T31* gas flow 30 l/min and temperature 31 °C, *HF60-T31* gas flow 60 l/min and temperature 31 °C, *HF30-T37* gas flow 30 l/min and temperature 37 °C, *HF60-T37* gas flow 60 l/min and temperature 37 °C

Patient comfort was significantly higher during steps performed at the lower temperature (HF30-T31 and HF60-T31) in comparison with the higher temperature (HF30-T37 and HF60-T37) (*p* < 0.0001 by GLIMMIX; see Fig. 1a legend for post hoc comparisons). On the contrary, comfort was not affected by flow values (Fig. 1a).

### Effects of flow and temperature on comfort in patients with FiO_2_ ≥ 45%

A comparison of baseline characteristics in the subgroups of patients requiring FiO_2_ < 45% vs FiO_2_ ≥ 45% [16] is reported in Table 1. Significant baseline differences were reported for FiO_2_, SpO_2_/FiO_2_, respiratory rate, and Borg score with worse values in the more hypoxemic patients, and these might confirm accurate stratification of AHRF severity by the chosen FiO_2_ threshold.

In both subgroups, lower temperature (HF30-T31 and HF60-T31 vs HF30-T37 and HF60-T37) was still associated with higher comfort, but, at variance from the whole population and the other subgroup, higher HFNC flow significantly improved comfort in patients with higher FiO_2_ (HF60-T31 and HF60-T37 vs HF30-T31 and HF30-T37, *p* < 0.001 by GLIMMIX; see figure legend for post hoc comparisons) (Fig. 1b).

### Optimum HFNC settings for patient comfort

In the whole population, on average, comfort during HFNC was elevated in all study phases (median 3 (IQR 2–4), mean 3.2 ± 1.4); at the patient level, fair to high comfort (i.e., ≥ 3) was always achieved in at least one phase. Four patients (10%) reached 3 as the maximum comfort level (two patients during HF30-T31, one patient during HF30-T37, one patient during HF60-T31, no patient during HF60-T37), 11 (27.5%) reached a level of 4 (four patients during HF30-T31, five patients during HF30-T37, one patient during HF60-T31, one patient during HF60-T37), and 25 (62.5%) reached a maximum score of 5 (nine patients during HF30-T31, nine patients during HF30-T37, one patient during HF60-T31, six patients during HF60-T37).

**Fig. 2:**
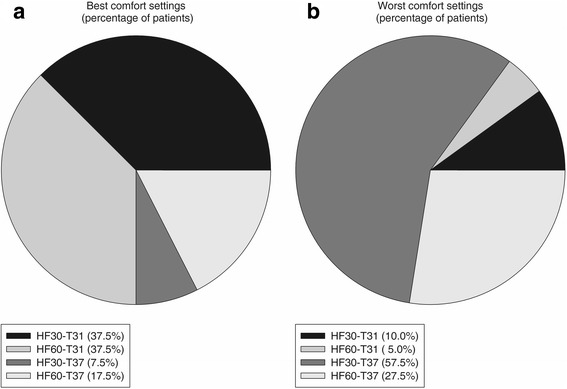
Best and worst comfort. Distribution of “best comfort settings” (**a**) and “worst comfort settings” (**b**) expressed as percentage of patients reporting highest or lowest comfort value in that particular study phase (see text for detailed description). *HF30-T31* gas flow 30 l/min and temperature 31 °C, *HF60-T31* gas flow 60 l/min and temperature 31 °C, *HF30-T37* gas flow 30 l/min and temperature 37 °C, *HF60-T37* gas flow 60 l/min and temperature 37 °C

“Best comfort settings” were HF30-T31 in 15 patients (37.5%), HF30-T37 in 15 patients (37.5%), HF60-T31 in three patients (7.5%), and HF60-T37 in seven patients (17.5%) (*p* = 0.01) (Fig. 2a). Conversely, “worse comfort settings” were HF30-T31 in four patients (10%), HF60-T31 in two patients (5%), HF30-T37 in 23 patients (57.5%), and HF60-T37 in 11 patients (27.5%) (*p* < 0.0001) (Fig. 2b).

### Effects of flow and temperature on vital signs and respiratory pattern

Among the collected variables, higher flow improved oxygenation (*p* < 0.0001 by GLIMMIX; see table legend for post hoc comparisons), while higher temperature was associated with increased heart rate (*p* = 0.021 by GLIMMIX; see table legend for post hoc comparisons) (Table 2). However, from a clinical point of view, changes in vital signs induced by different HFNC settings were only marginally relevant.

**Table 2:** Comparison of vital signs during each study phase (see text for details)

Variable	HF30-T31 (*N* = 40)	HF60-T31 (*N* = 40)	HF30-T37 (*N* = 40)	HF60-T37 (*N* = 40)	*p* value, GLIMMIX
Systolic arterial pressure (mmHg)	122 (112–136)	121 (109–135)	124 (109–138)	120 (111–135)	0.854
Diastolic arterial pressure (mmHg)	64 (59–70)	63 (56–70)	65 (53–70)	65 (60–70)	0.731
Heart rate (bpm)	88 ± 15	87 ± 15	90 ± 15^‡^	89 ± 15	0.02**
SpO_2_ (%)	96 (94–99)	98 (96–100)^†^	96 (95–99)^§^	98 (96–99)^†||^	< 0.0001**
Respiratory rate (bpm)	22 (18–29)	22 (19–25)	23 (19–29)	23 (19–26)	0.174
Borg scale	3 (2–5)	3 (1–5)	4 (2–5)	3 (2–5)	0.557
SpO_2_/FiO_2_	238 (192–246)	240 (196–248)^†^	240 (190–248)^‡^	240 (196–248)*	< 0.001**

*FiO*_*2*_ inspired oxygen fraction, *GLIMMIX* generalized linear mixed model, *HF30-T31* gas flow 30 l/min and temperature 31 °C, *HF60-T31* gas flow 60 l/min and temperature 31 °C, *HF30-T37* gas flow 30 l/min and temperature 37 °C, *HF60-T37* gas flow 60 l/min and temperature 37 °C, *SpO*_*2*_ peripheral oxygen saturation

Post hoc comparisons: *p < 0.01 vs HF30-T31; ^†^*p* < 0.001 vs HF30-T31; ^‡^*p* < 0.05 vs HF60-T31; ^§^*p* < 0.001 vs HF60-T31; ^||^*p* < 0.001 vs HF30-T37

**Data significant at *p* < 0.05

## Discussion

The main findings of our study can be summarized as follows: in AHRF patients undergoing HFNC, comfort assessed by Visual Numeric Scale was higher at lower temperature, regardless of flow rate; in the subgroup of more severe patients (i.e., those with clinical FiO_2_ ≥ 45%), both lower temperature and higher flow were associated with improved comfort; and HFNC settings associated with best and worst comfort presented large variability at the individual patient level.

In humans, the alveolar membrane is a dead-ended structure reached only by isothermal (100% relative humidity and 44 mg/l H_2_O absolute humidity) and body-warm (37 °C) air. Thus, the respiratory support respecting this condition the most should be associated with increased comfort and, potentially, with decreased unphysiologic mechanisms (e.g., inflammation, reduced immunity, altered airway patency) [18–23]. The HFNC, unlike cool and anhydrous conventional oxygen therapy, can deliver to the alveoli an air–oxygen moisturized and heated blend. In this study, we measured patient comfort during various HFNC settings, as comfort could represent a balanced synthesis of various physiologic mechanisms, potentially being a patient-level outcome per se [9, 10]. Previous physiologic studies in patients undergoing non-invasive respiratory support assessed comfort by the same method as used in this study (i.e., VNS) [24, 25]. Comfort during non-invasive support could be particularly relevant for improving tolerance. Indeed, an ongoing randomized controlled trial on a musical intervention in ICU patients on non-invasive ventilation has comfort evaluated by VNS as a primary endpoint [26]. Finally, a recent post hoc analysis of the FLORALI study identified poor comfort as the only predictor of intubation in patients on HFNC within 1 h from the start, thus suggesting a link between comfort and improved hard clinical outcomes [27]. Interestingly, in all of these studies [1, 24, 25, 27] dyspnea was independently assessed by the Borg scale as we did. Relief of dyspnea is only one component of improved comfort, and discomfort can still be present at relatively low dyspnea scores, its determinants being wider and more holistic (e.g., including also physiologic and sensorial factors).

Our findings that comfort is higher at T31 than at T37 independently from the set flow rate might suggest that a series of negative physiologic (e.g., unbalanced water retention) and psychosomatic (e.g., excessive heating of the nostrils) signals might prevail over the advantage of maximum humidity. However, since during use of the HFNC the heating and moisturizing function of the upper airways is preserved, inspired gases at 31 °C with full humidification should already prevent airway dryness and associated lesions. Hence, starting the HFNC at lower temperature (and eventually increasing it with time) may be a reasonable clinical approach to exploit the positive clinical outcomes deriving from higher tolerance, longer application of the non-invasive support, and improved comfort per se.

Equally complex are the effects of flow rate on comfort, due to the potential interplay between mechanical, chemical, and psychological stimuli [28, 29]. Previous studies showed that higher HFNC flow allows a more reliable correspondence between set and alveolar FiO_2_ and obtains higher PEEP effects [8, 14]. Our finding that, in the whole population, comfort at lower flow did not differ significantly from higher flow might indicate either a low patient severity (with relatively low inspiratory flow) or a prevalence of biochemical stimuli and sensation of airway dryness over those deriving from more effective mechanical displacement of the respiratory system.

In more severe patients clinically requiring higher FiO_2_ levels, better comfort was reached at higher flow rates (60 l/min) and this might suggest that more effective correction of hypoxemia and/or improved lung mechanics [7, 8] might have prevailed at higher flow as determinants of comfort. As a clinical reference, higher flows could be recommended in more severe patients requiring higher FiO_2_ as this could couple improved physiology [8, 14] with higher comfort.

The finding that the “best and worst comfort” was reached at different combinations of flow and temperature in the individual patient might suggest that HFNC is yet another ICU treatment that could benefit from personalized rather than average standardized settings [30]. AHRF is a highly heterogeneous syndrome with regards to etiology (e.g., trauma vs infection), lung mechanics (higher vs lower compliance), and systemic involvement (number of organ failures) [31] and it seems physiologically sound that the same “average” treatment should not be applied to all patients. As in the acute respiratory distress syndrome, where PEEP, tidal volume, fluid management, and extracorporeal CO_2_ removal might have largely different effects based on patient subphenotype [32–35], HFNC settings might need to be applied in an individualized fashion. To this end, our findings might suggest that comfort could be the bedside endpoint to personalize HFNC titration, but this should be validated in larger studies.

Our study has some limitations. First, its primary aim was to assess patient comfort, which might be viewed only as a psychologic secondary outcome. However, patients experience a sense of relief when their physical and psychologic comfort needs are met [9, 10], and comfort might be an accurate multifaceted method to estimate and improve the effectiveness of non-invasive respiratory support [24–26]. Moreover, pilot data suggest a link between poor comfort early during application of HFNC and subsequent intubation [27]. Second, the results derive from a short-time observation (20 min for each phase) and they should be confirmed by more extensive surveillance. Third, we tried to precisely define AHRF but our population was likely highly heterogeneous in terms of etiology, inflammation activation, and derangement of respiratory mechanics. Fourth, the more severe subgroup was identified by clinically set FiO_2_, which might have some individual discretion. However, clinical FiO_2_ was somehow standardized to obtain acceptable SpO_2_ and could also reflect a more global evaluation of the patient’s severity by the attending physician; a previous study showed high discriminatory power of FiO_2_ for ARDS severity [16]; and objective assessment of AHRF severity in non-intubated patients is challenging as accepted measures such as intrapulmonary shunt or respiratory system compliance are lacking. Fifth, we did not explore midway settings but only extremes of temperature and flow; for this reason, it is difficult to understand whether the correlation between comfort and temperature is linear or characterized by a steep threshold change.

## Conclusions

Set temperature during use of the HFNC seems to significantly impact the comfort of AHRF patients: for equal flow, lower temperature could be more comfortable. Higher flow does not decrease comfort: on the contrary, in the subgroup of more severely hypoxemic patients, higher flow improves comfort. At the individual patient level, high variability exists in the settings associated with highest and lowest comfort, and the HFNC might need personalized titration. To this end, patient comfort might already represent a smart and crafty indicator to guide settings of non-invasive respiratory support by HFNC.

## Abbreviations

AHRF: Acute hypoxemic respiratory failure
BMI: Body mass index
CO_2_: Carbon dioxide
CT: Computed tomography
FiO_2_: Inspired oxygen fraction
GLIMMIX: Generalized linear mixed model
HF30-T31: Gas flow 30 l/min and temperature 31 °C
HF30-T37: Gas flow 30 l/min and temperature 37 °C
HF60-T31: Gas flow 60 l/min and temperature 31 °C
HF60-T37: Gas flow 60 l/min and temperature 37 °C
HFNC: High-flow nasal cannula
ICU: Intensive care unit
IQR: Interquartile range
NIPPV: Non-invasive positive pressure ventilation
PaO_2_: Partial oxygen pressure
PEEP: Positive end expiratory pressure
P-SILI: Patient self-inflicted lung injury
SAPS: Simplified Acute Physiology Score
SOFA: Sepsis-related Organ Failure Assessment
